# Fenofibrate attenuates renal lipotoxicity in uninephrectomized mice with high-fat diet-induced obesity

**DOI:** 10.1590/2175-8239-JBN-2023-0148en

**Published:** 2024-09-09

**Authors:** Barbara Bruna Abreu Castro, Petrus Ferreira Reno, Bianca Fatima Pereira, Kaique Arriel, Fabiana Bastos Bonato, Fernando Antonio Basile Colugnati, Marcos Antonio Cenedeze, Niels Olsen Saraiva-Camara, Helady Sanders-Pinheiro

**Affiliations:** 1 Universidade Federal de Juiz de Fora, Centro de Biologia da Reprodução, Núcleo de Experimentação Animal, Laboratório de Nefrologia Experimental, Juiz de Fora, MG, Brazil.; 2 Universidade Federal de Juiz de Fora, Divisão de Nefrologia, Núcleo Interdisciplinar de Estudos e Pesquisas em Nefrologia, Juiz de Fora, MG, Brazil.; 3 Universidade Federal de São Paulo, Divisão de Nefrologia, Laboratório de Imunologia Clínica e Experimental, São Paulo, SP, Brazil.; 4 Universidade de São Paulo, Instituto de Ciências Biomédicas, Departamento de Imunologia, Laboratório de Imunologia de Transplantes, São Paulo, SP, Brazil.

**Keywords:** Lipid Metabolism Disorders, Obesity, Nephrectomy, PPAR alpha, Fibroblast Growth Factors

## Abstract

**Introduction:**

The objective of this study was to investigate the role of fenofibrate, a peroxisome proliferator-activated receptor-α agonist, in obesity-induced kidney damage (lipotoxicity) in mice with uninephrectomy.

**Methods:**

C57BL/6 mice underwent uninephrectomy and sham surgeries and were fed normocaloric or high-fat diets. After 10 weeks, obese mice were administered 0.02% fenofibrate for 10 weeks. Kidney function and morphology were evaluated, as well as levels of inflammatory and fibrotic mediators and lipid metabolism markers.

**Results:**

High-fat diet-fed mice developed characteristic obesity and hyperlipidemia, with subsequent renal lipid accumulation and damage, including mesangial expansion, interstitial fibrosis, inflammation, and proteinuria. These changes were greater in obese uninephrectomy mice than in obese sham mice. Fenofibrate treatment prevented hyperlipidemia and glomerular lesions, lowered lipid accumulation, ameliorated renal dysfunction, and attenuated inflammation and renal fibrosis. Furthermore, fenofibrate treatment downregulated renal tissue expression of plasminogen activator inhibitor-1, monocyte chemoattractant protein-1, and local expression of fibroblast growth factor-21.

**Conclusion:**

Peroxisome proliferator-activated receptor-α activation by fenofibrate, with subsequent lipolysis, attenuated glomerular and tubulointerstitial lesions induced by renal lipotoxicity, thus protecting the kidneys of uninephrectomy mice from obesity-induced lesions. The study findings suggest a pathway in the pharmacological action of fenofibrate, providing insight into the mechanisms involved in kidney damage caused by obesity in kidney donors.

## Introduction

Obesity and metabolic syndrome, which are independent risk factors for chronic kidney disease (CKD), are associated with hyperlipidemia, adipocytokine level changes, and increased oxidative stress, inflammation, apoptosis, and renal tissue fibrosis^1,2^. The recruitment of inflammatory cells in the kidney leads to the production of reactive oxygen species, and changes renal hemodynamics, inducing plasminogen activator inhibitor-1 (PAI-1) expression^3^. Additionally, renal lipotoxicity caused by excess triglycerides (TG) or fatty acids activates the production of inflammatory cytokines and the expression of monocyte chemoattractant protein 1 (MCP-1) in kidney tissue^1,4,5^.

Renal lipotoxicity in the remaining kidney of obese kidney donors can accelerate CKD development and progression, although the mechanisms are unclear^6^. In mice, uninephrectomy (UNX) followed by high-fat diet (HFD) causes mesangial expansion, glomerulosclerosis, and interstitial fibrosis in the remnant kidney^7^. Subsequent obesity due to HFD leads to more severe changes and increases the expression of genes associated with lipid metabolism and transmembrane lipid transport^7^.

Therapies aimed at decreasing serum and tissue TG levels, ROS production, and inflammation have been investigated in animal obesity models^8^. Fenofibrate (FF), a peroxisome proliferator-activated receptor (PPAR)-α agonist, protects against HFD-induced kidney damage in animal models^1,9^. Moreover, FF improved interleukin (IL)-6-dependent renal anti-inflammatory pathways in a UNX animal model, although the animals were not overweight^10^. PPAR-α regulates intracellular lipid stores^1,6,11^ and the expression of fibroblast growth factor-21 (FGF-21) in the liver, and can also be expressed in adipose tissues, heart, and kidney^12^. A study examining the effects of FF in an obesity model revealed that FGF-21 is a mediator in the protection pathway against kidney damage^13^. Pharmacological activation of PPAR-α in the kidney may exert therapeutic effects through lipolysis, thus attenuating the deleterious effects of lipotoxicity^2,11^.

The aim of this study was to determine the role of FF in renal lipotoxicity associated with decreased renal mass using a UNX mouse model with HFD-induced obesity to mimic kidney donors who become obese.

## Methods

### Experimental Protocol

**Figure 1:**
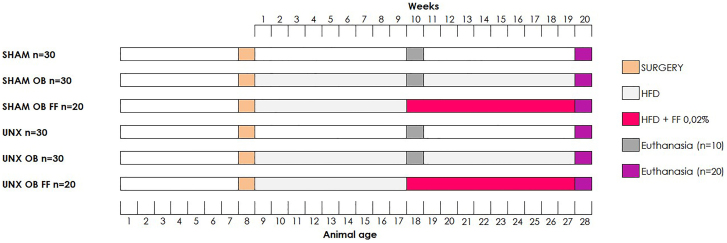
Experimental design. Abbreviations – SHAM: simulated surgery; OB: obese; FF: fenofibrate; UNX: uninephrectomy; HFD: high-fat diet.

Eight-week-old male C57BL/6 mice weighing an average of 24 ± 1.6 g were subjected to SHAM or UNX procedures (Figure 1). In the SHAM group, the left kidney was decapsulated without being removed^14^; in the UNX group, the left kidney was removed through an abdominal incision^15^. Obesity was induced by the administration of a high-fat diet with an energy intake of 5,625 kcal/kg (Pragsoluções SA, Jaú, Brazil). The caloric composition of the HFD was: 28.1% protein, 37.3% fat, and 27.2% carbohydrate (60% energy from total fat)^7^ (Table S1). The caloric composition of the normocaloric diet was: 22% protein, 5% fat, and 57% carbohydrate; gross energy 3,860 kcal/kg (Nuvilab, Curitiba, Brazil). FF (Sigma-Aldrich, St. Louis, USA) was added to the diet (0.02%, 20 mg/kg/d) from weeks 10 to 20^2,6,9^.

At 10 and 20 weeks, mice were anesthetized by intraperitoneal injection of xylazine (10 mg/kg) and ketamine (90 mg/kg) (Sigma-Aldrich). Blood samples were collected via cardiac puncture, then the mice were euthanized by diaphragm rupture and the right kidney was removed for analysis.

All animal procedures were carried out in accordance with the ethical standards of the Animal Ethics Committee of the Federal University of Juiz de Fora (IRB approval number 046/2018).

### Dietary Intake and Obesity Changes

Feed intake and total animal weight were measured (g) every 4 weeks (Figure S1) and mean daily intake was calculated. As a measure of changes induced by HFD, we assessed the total weight, weight gain, total fat, and the Lee index at 10 and 20 weeks. We considered total fat to be the retroperitoneal and epididymal fat pads removed and weighed at euthanasia^7,14,16^. Nasoanal length was measured (cm) and used to calculate the Lee index^17^.

### Blood and Urine Analysis

Mice were kept in metabolic cages for 24 h for urine collection, and 24-h proteinuria was determined (mg/24 h) using a Sensiprot Liquiform kit (Labtest, Lagoa Santa, Brazil). Serum creatinine levels were measured using the Creatinina K Liquiform assay (Labtest) to calculate creatinine clearance. Creatinine clearance was calculated as 24-hour urinary creatinine excretion (mg) divided by serum creatinine (mg/dL), times 1440 (U×V/P×1440) and expressed in mL/min^7,14^. Serum TG and total cholesterol levels were measured using a Cobas analyzer (Roche Diagnostics, Basel, Switzerland) at week 20.

### Lipid Extraction and Kidney Lipid Content

Lipids were extracted by homogenizing 35 mg renal tissue with 500 μL chloroform/methanol and water solution, followed by centrifugation at 3,000 rpm for 5 min. The bottom layer was transferred to a new tube. After evaporating the liquid, 100 μL isopropyl alcohol was added to the sample for lipid solubilization^1^. TG and cholesterol levels were determined using a colorimetric Liquiform Cholesterol and Triglycerides enzymatic test (Labtest) at weeks 10 and 20.

### Histological Analysis

Half of the right kidney was fixed in 10% formalin for histological analysis^7,14,18,19,20^. Renal tissue samples were obtained from five animals per treatment group. Sections (5 μm) were stained with hematoxylin-eosin (H&E) and sirius red (Sigma-Aldrich). The slides were examined and photographed using an Axio Scope A1 microscope (Carl Zeiss, Göttingen, Germany) coupled to a digital microscope camera using AmScope MU1000 system software (AmScope, Irvine, TX, USA).

Semi-quantitative and quantitative analyses of mesangial expansion were done. To evaluate mesangial expansion semi-quantitatively, slides from 5 animals from each group were analyzed. Ten glomeruli with vascular poles per slide were photographed at 400× magnification. Each glomerulus was qualitatively analyzed for mesangial expansion, defined as the mesangial space exceeding the width of two mesangial cells by at least two glomerular lobes and was classified as “Absence” or “Presence” of mesangial expansion^18,19^. The results were expressed as the percentage of glomeruli with mesangial expansion per slide.

Quantitative analysis was performed using ImageJ 1.52n software (National Institutes of Health, Bethesda, MD, USA). Slides from 5 animals from each group were analyzed. Ten glomeruli with vascular poles per slide were photographed at 400× magnification, which corresponds to an area of 0.045 mm^2^. In each photograph, the entire area occupied by the glomerular tuft was marked. Measurement of the area occupied in the image provided numerical values expressed in pixels. This variable was named glomerular area^21^.

Renal fibrosis was quantified via sirius red staining under polarized light^20^. Ten photomicrographs were taken per slide at 20× magnification. Bright red areas were quantified using ImageJ 1.52n software. The results were expressed as the percentage of interstitial area of fibrosis per cortex/total cortical area. Subsequently, the mean of the positive staining area was calculated for each slide^21^.

### Adipocytokines and PAI-1 Prefibrotic Factor

Approximately 35 mg of tissue was macerated in 1 mL RIPA lysis buffer at 4°C, and protein concentration was measured using the Micro BCA Protein Assay kit (Thermo Fisher Scientific, Waltham, MA, USA). The concentrations of adiponectin, leptin, and PAI-1 prefibrotic factor present in the sample were analyzed using the MILLIPLEX MAP Mouse Adipocyte Magnetic Panel – Endocrine Multiplex Assay (Merck, Barueri, Brazil). The test was performed using the Bio-Plex 200 System with Bio-Plex Manager software version 5.0 (Bio-Rad, Hercules, CA, USA)^22^.

### Inflammatory and Fibrotic Mediators Evaluated Through Quantitative Real-Time PCR

The TaqMan amplification system (Applied Biosystems, Branchburg, NJ, USA) was used to conduct quantitative real-time PCR (qRT-PCR) with the thermal cycler 7500 Real Time PCR System (Applied Biosystems, Singapore). All samples were analyzed in triplicate. A comparative relationship between reaction cycles (CT) was used to determine the expression of the target gene in relation to the control gene hypoxanthine-guaninephosphoribosyltransferase^23^. Primers and probes synthesized for IL-6, IL-1β, MCP-1, IFN-γ, and FGF-21 were used to evaluate the effect of FF on the inflammatory profile (Assays-On-Demand Gene Expression products; Applied Biosystems, Foster City, CA, USA) (Table S2). CT values of target genes were normalized to their respective control genes for each sample, and the resulting value was used to demonstrate the relative expression of target genes through the 2-ΔΔCT method, as described previously^24^.

### Statistical Analysis

Kolmogorov-Smirnov and Shapiro-Wilk tests were used to evaluate the distribution of variables. Homogeneity of variance was verified using Levene’s test. The reproducibility of lesions in the experimental models (obesity and UNX) was evaluated at week 10 using one-way analysis of variance (ANOVA), followed by Bonferroni’s post-hoc test. The effects of UNX, diet, and FF treatment on the evaluated parameters was verified at week 20 using a general linear model to compare groups using two-way ANOVA, followed by Bonferroni’s post-hoc test. Some variables were transformed to log 10 values to obtain normal distribution and variance homogeneity. The Kruskal-Wallis nonparametric test followed by the Mann-Whitney test was used for variables that did not meet the normality and homogeneity criteria for comparison between groups. Statistical significance was set at P < 0.05. Statistical analysis was performed using SPSS version 15.0 software (SPSS Inc., Chicago, IL, USA). Data used in the analysis are available on request at Open Science Framework, https://osf.io/s3j9a.

## RESULTS

### Evaluation of UNX and Obesity Model Mice at Week 10

Obesity with renal dysfunction model was successfully induced by HFD after 10 weeks (Table 1). The UNX group exhibited higher renal weights than the SHAM group, without loss of renal function (Table 1). The UNX OB group had lower dietary intake, greater fat accumulation, higher Lee index values, and greater proteinuria and hyperfiltration than the UNX group (Table 1).

**Table 1:** Metabolic and renal function parameters of mice subjected to SHAM or UNX surgeries and fed normocaloric or high-fat diets for 10 weeks. data are presented as the mean ± standard deviation

Parameters	10 weeks			
**Group (N)**	**SHAM (N = 9)**	**SHAM OB (N = 10)**	**UNX (N = 8)**	**UNX OB (N = 8)**
Intake (g/d)	3.6 ± 0.7	2.2 ± 0.3^a^	4.2 ± 0.7^b^	2.6 ± 0.3^a c^
Total weight (g)	27.2 ± 1.1	29.8 ± 3.3	25.2 ± 0.9^b^	27.7 ± 2.2
Weight gain (g)	3.0 ± 2.0	6.5 ± 1.9^a^	3.4 ± 1.8^b^	4.4 ± 2.1
Total fat (g)	0.41 ± 0.17	1.46 ± 0.66^a^	0.19 ± 0.08^b^	1.03 ± 0.38^a c^
Kidney weight (g)	0.17 ± 0.01	0.15 ± 0.01	0.20 ± 0.03^a b^	0.20 ± 0.01^b^
Lee index (g/cm^3^)	310 ± 6	318 ± 6^a^	306 ± 5^b^	308 ± 6^b^
**Group (N)**	**SHAM (N = 3)**	**SHAM OB (N = 3)**	**UNX (N = 3)**	**UNX OB (N = 3)**
Proteinuria (mg/24 h)	2.1 ± 1.2	9.0 ± 2.6^a^	1.0 ± 1.1^b^	10.9 ± 1.5^a c^
Serum creatinine (mg/dL)	1.0 ± 0.1	0.9 ± 0.1	0.9 ± 0.1	0.9 ± 0.1
Creatinine clearance (mL/min)	0.06 ± 0.04	0.25 ± 0.09	0.04 ± 0.03^b^	0.34 ± 0.07^a c^
**Group (N)**	**SHAM (N = 5)**	**SHAM OB (N = 5)**	**UNX (N = 5)**	**UNX OB (N = 4)**
Kidney TG (mg/g tissue)	2.91 ± 0.87	4.06 ± 0.25	3.29 ± 0.25	4.56 ± 1.40
**Group (N)**	**SHAM (N = 4)**	**SHAM OB (N = 4)**	**UNX (N = 4)**	**UNX OB (N = 4)**
Kidney cholesterol (mg/g tissue)^log^	3.60 ± 0.25	3.51 ± 0.24	3.83 ± 0.24	4.67 ± 1.18

Abbreviations – SHAM: simulated surgery; OB: obese; UNX: uninephrectomy; HFD: high-fat diet; TG: triglycerides.

Notes – ^log^Variables were transformed to log 10 for analysis. Groups were compared using ANOVA followed by the Bonferroni’s *post-hoc* test.

^a^ *P* < 0.05 vs. SHAM,

^b^ *P* < 0.05 vs. SHAM OB,

^c^ *P* < 0.05 vs. UNX.

### Evaluation of Obesity Model After FF Treatment

At the end of the experiment, the SHAM OB and UNX OB groups (HFD-fed groups) presented with lower dietary intake and greater obesity-associated changes than the SHAM and UNX groups (Table 2).

**Table 2:** Metabolic parameters, lipid profiles, levels of adipocytokines and inflammatory markers, and pai-1 expression in mice subjected to sham and unx surgeries, fed norm

Parameters	20 weeks					
Group (N)	SHAM (N = 18)	SHAM OB (N = 19)	SHAM OB FF (N = 17)	UNX (N = 19)	UNX OB (N = 18)	UNX OB FF (N = 17)
Intake (g/d)	3.48 ± 0.45	2.51 ± 0.24^a^	2.68 ± 0.34^a^	3.47 ± 0.42	2.51 ± 0.30^c^	2.69 ± 0.55^c^
Total weight (g)	27.2 (24.5 – 30.0)	35.5 (29.5 – 46.5)^a^	33.5 (26.0 – 44.0)^a^	28.0 (25.0 – 31.5)	33.2 (25.0 – 46.0)^c^	32.0 (26.5 – 47.5)^c^
Weight gain (g)^log^	5.67 ± 1.12	13.53 ± 4.21^a^	9.55 ± 5.35^a^	5.24 ± 2.06	10.81 ± 5.65^c^	10.95 ± 6.22^c^
Total fat (g)	0.35 (0.17 – 0.51)	2.49 (0.76 – 3.30)^a^	1.56 (0.47 – 2.56)^a b^	0.34 (0.20 – 0.71)	1.62 (0.35 – 3.38)^c^	2.00 (0.70 – 3.24)^c^
Kidney weight (g)^log^	0.17 ± 0.02^c d^	0.16 ± 0.02^c d^	0.18 ± 0.01^c d^	0.23 ± 0.02^a b^	0.22 ± 0.02^a b^	0.22 ± 0.02^a b^
Lee index (g/cm^3^)	305 (296 – 321)	319 (305 – 342)^a^	313 (299 – 328)^a b^	302 (292 – 312)	315 (292 – 332)^c^	318 (305 – 344)^c^
**Group (N)**	**SHAM** **(N = 9)**	**SHAM OB** **(N = 10)**	**SHAM OB FF** **(N = 10)**	**UNX** **(N = 10)**	**UNX OB** **(N = 10)**	**UNX OB FF** **(N = 9)**
Serum TG (mg/dL)^log^	89.9 ± 25.4	102.7 ± 37.2	58.0 ± 17.1^a b^	79.5 ± 14.6	113.9 ± 51.4	79.4 ± 21.2^c d^
**Group (N)**	**SHAM** **(N = 9)**	**SHAM OB** **(N = 10)**	**SHAM OB FF** **(N = 10)**	**UNX** **(N = 9)**	**UNX OB** **(N = 10)**	**UNX OB FF** **(N = 9)**
Serum cholesterol (mg/dL)^log^	83.4 ± 11.2	185.0 ± 15.0^a^	213.9 ± 14.5^a b^	95.2 ± 8.5^a^	194.5 ± 35.1^c^	229.7 ± 17.1^c d^
**Group (N)**	**SHAM** **(N = 11)**	**SHAM OB** **(N = 11)**	**SHAM OB FF** **(N = 11)**	**UNX** **(N = 11)**	**UNX OB** **(N = 12)**	**UNX OB FF** **(N = 11)**
Adiponectin (pg/mg protein)	7.12 ± 1.51	4.93 ± 1.71^a^	6.26 ± 1.02^a^	7.08 ± 1.54	5.45 ± 1.22^c^	5.75 ± 1.06^c^
Leptin (pg/mg protein)^log^	0.08 ± 0.02	0.17 ± 0.05^a^	0.14 ± 0.06^a b^	0.09 ± 0.03	0.30 ± 0.15^c^	0.20 ± 0.07^c d^
PAI-1 (pg/mg protein)	0.011 ± 0.002	0.015 ± 0.005	0.012 ± 0.002^b^	0.015 ± 0.005	0.017 ± 0.006	0.011 ± 0.003^d^
**Group (N)**	**SHAM** **(N = 10)**	**SHAM OB** **(N = 10)**	**SHAM OB FF** **(N = 10)**	**UNX** **(N = 10)**	**UNX OB** **(N = 10)**	**UNX OB FF** **(N = 10)**
IL1-B (2^△△CT^)	0.71 (0.46 – 1.29)	3.49 (2.44 – 4.42)^a^	0.74 (0.49 – 1.68)^b^	3.48 (2.53 – 7.08)	3.78 (2.01 – 10.90)	0.64 (0.37 – 1.00)^c d^
MCP-1 (2^△△CT^)^log^	1.25 ± 0.58	2.48 ± 0.96^a^	1.10 ± 0.46^a b^	2.71 ± 0.61	3.41 ± 1.36^c^	0.99 ± 0.23^c d^
IFN-g (2^△△CT^)	0.84 ± 0.19	2.21 ± 0.39^a^	1.34 ± 0.21^b^	1.96 ± 0.35	2.99 ± 0.53^c^	1.77 ± 0.37^d^
**Group (N)**	**SHAM** **(N = 8)**	**SHAM OB** **(N = 6)**	**SHAM OB FF** **(N = 9)**	**UNX** **(N = 3)**	**UNX OB** **(N = 7)**	**UNX OB FF** **(N = 10)**
IL -6 (2^△△CT^)^log^	0.54 ± 0.34	1.66 ± 0.83^a^	1.15 ± 0.93^b^	0.83 ± 0.17	2.62 ± 1.88^c^	0.51 ± 0.34^d^
**Group (N)**	**SHAM** **(N = 9)**	**SHAM OB** **(N = 10)**	**SHAM OB FF** **(N = 9)**	**UNX** **(N = 10)**	**UNX OB** **(N = 8)**	**UNX OB FF** **(N = 8)**
FGF -21 (2^△△CT^)^log^	0.71 ± 0.50	5.37 ± 3.60^a^	0.69 ± 0.51^a b^	7.28 ± 3.93	10.67 ± 10.44^c^	0.40 ± 0.38^c d^

Abbreviations – SHAM: simulated surgery; OB: obese; FF: fenofibrate; UNX: uninephrectomy; TG: triglycerides; IL -1β: interleukin-1β; IL -6: interleukin-6; MCP-1: monocyte chemoattractant protein-1; IFN-γ: interferon-γ; FGF-21: fibroblast growth factor-21; PAI-1: inducing plasminogen activator inhibitor-1.

Notes – ^log^Variables transformed to log 10 for analysis. Groups were compared using two-way ANOVA followed by Bonferroni’s *post hoc* test or by the Kruskal–Wallis test followed by the Mann–Whitney test.

^a^ *P* < 0.05 vs. SHAM;

^b^ *P* <0.05 vs. SHAM OB;

^c^ *P* < 0.05 vs. UNX,

^d^ *P* < 0.05 vs. UNX OB.

The SHAM OB FF group exhibited lower fat accumulation and Lee index values compared to the SHAM OB group (Table 2). In contrast, the UNX OB FF group displayed no difference in obesity parameters compared with the UNX OB group (Table 2). Furthermore, the UNX groups exhibited greater renal weights than the SHAM groups (Table 2). Treatment with FF had no effect on dietary intake in either the SHAM OB FF or UNX OB FF groups, although it prevented fat accumulation in the SHAM OB FF group, indicated by a lower Lee index value than the SHAM group (Table 2).

### Lipid Profile After FF Treatment

FF has been shown to reduce serum TG levels; therefore, we evaluated the lipid profile of the UNX OB model. Although TG levels were not significantly higher in the SHAM OB and UNX OB groups, FF treatment reduced TG levels in the SHAM OB FF and UNX OB FF groups. Cholesterol levels did not change significantly after FF treatment (Table 2).

### Renal Function and Glomerulopathy after FF Treatment

**Figure 2:**
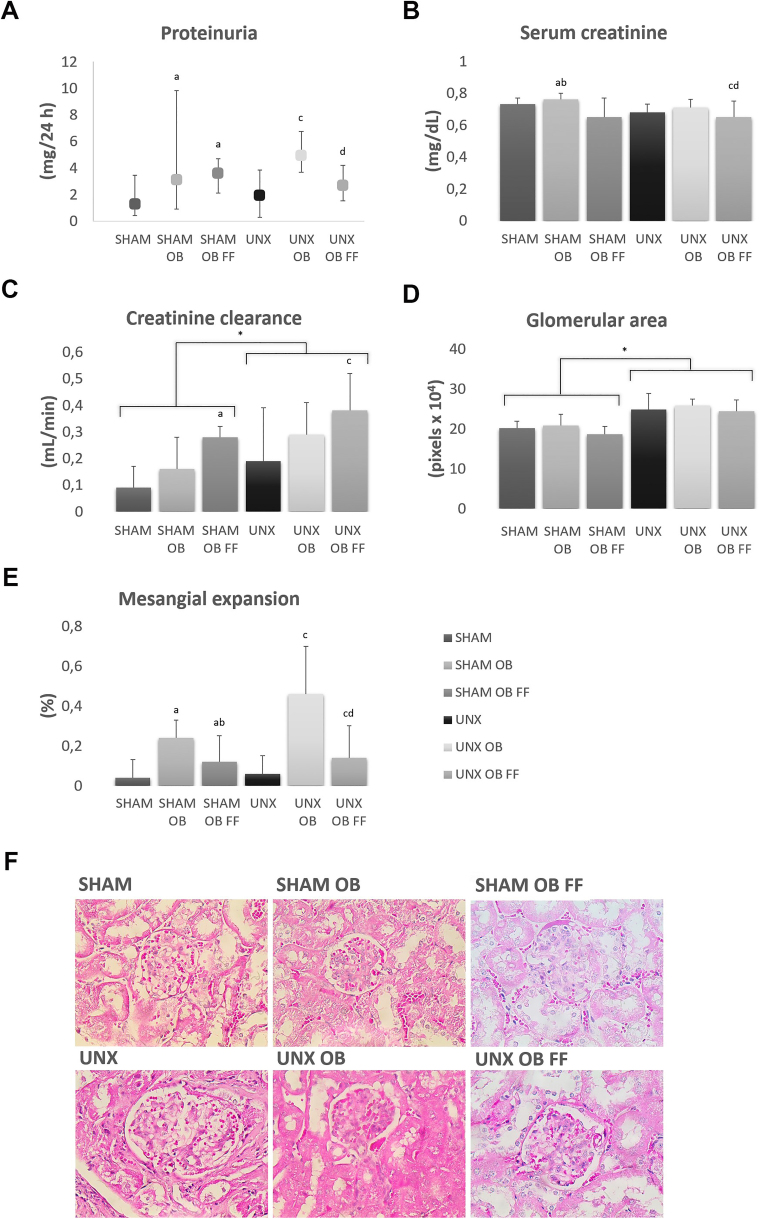
Evaluation of kidney function (a, b, c) and glomerular morphology (d, e, f: H&E, 400×) after obesity induction and FF treatment (10 weeks). Abbreviations – SHAM: simulated surgery; OB: obese; FF: fenofibrate; UNX: uninephrectomy; HE: hematoxylin–eosin. Notes – ^log^Variables transformed to log 10 for analysis. Groups were compared using two-way ANOVA followed by Bonferroni’s *post hoc* test or the Kruskal-Wallis test followed by the Mann-Whitney test. Data are presented as the mean ± standard deviation or median and minimum and maximum values. ^a^ *P* < 0.05 vs. SHAM; ^b^ *P* < 0.05 vs. SHAM OB; ^c^ *P* < 0.05 vs. UNX, ^d^ *P* < 0.05 vs. UNX OB.

Obesity induction in the SHAM OB and UNX OB groups was associated with increased proteinuria (Figure 2a). However, FF treatment attenuated the progression of lesions induced by obesity or lipotoxicity, indicated by reduced proteinuria in the UNX OB FF group (Figure 2a). Obesity did not increase serum creatinine levels (Figure 2b). Creatinine clearance was greater in the UNX, UNX OB, and UNX OB FF groups than in the SHAM, SHAM OB, and SHAM OB FF groups. Obesity caused hyperfiltration, which was not ameliorated with FF treatment (Figure 2c).

H&E staining of renal tissues revealed that the glomerular area increased in UNX groups regardless of obesity, which was unchanged by FF treatment (Figure 2d). The percentage of glomeruli with mesangial expansion in the SHAM OB and UNX OB groups was higher than that in the SHAM and UNX groups, respectively (Figure 2e). The SHAM OB FF and UNX OB FF groups displayed 50% and 70% reductions in mesangial expansion compared with the SHAM OB and UNX OB groups, respectively (Figure 2e and f).

### Lipid Deposits in Kidneys and Adipocytokine Levels After FF Treatment

**Figure 3:**
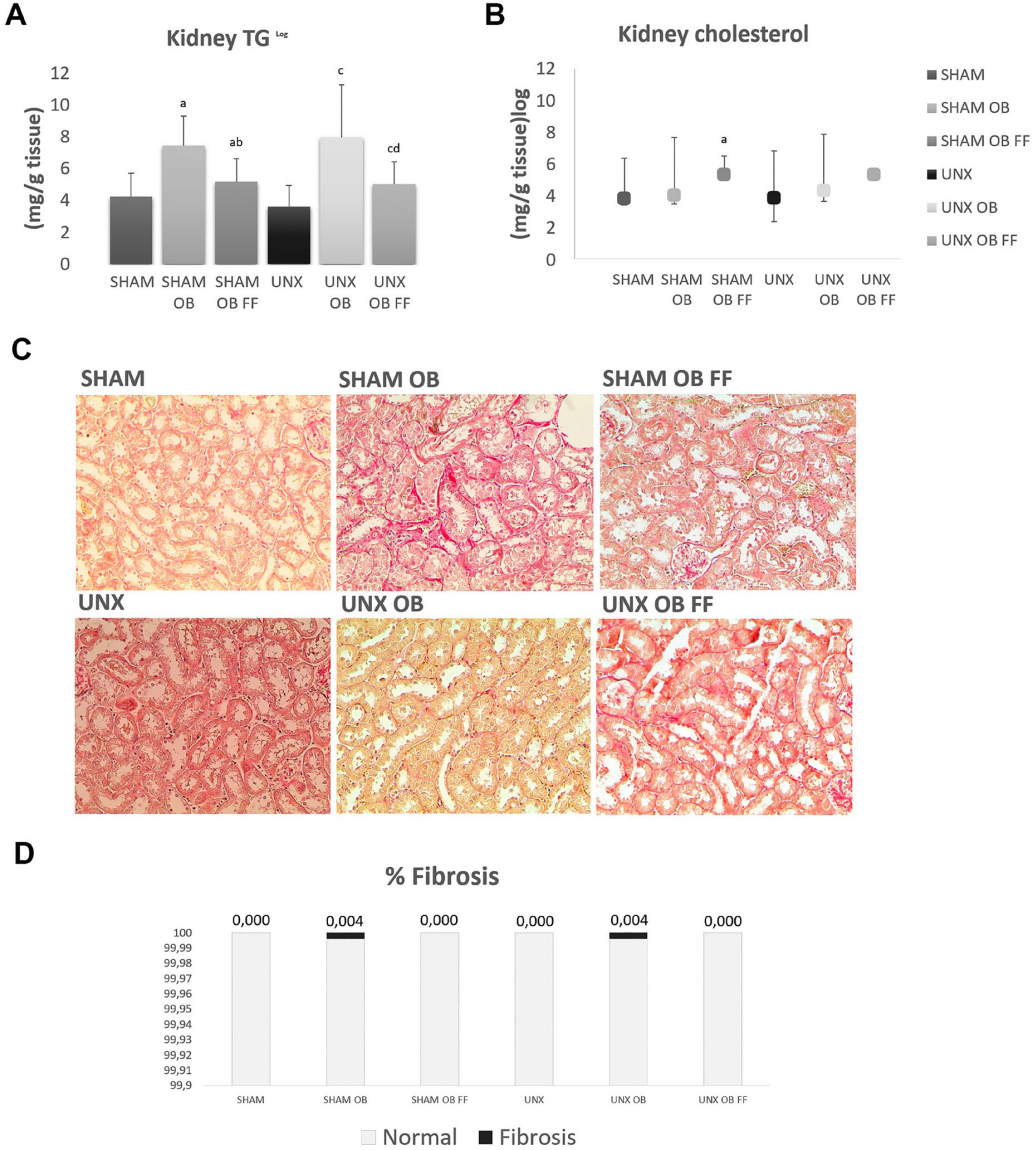
Lipid deposition values in kidney tissue (a, b) and renal interstitial fibrosis (c: Sirius red, 200×, D) after obesity induction and FF treatment (10 weeks). Abbreviations – SHAM: simulated surgery; OB: obese; FF: fenofibrate; UNX: uninephrectomy; TG: triglycerides. Notes – ^log^Variables transformed to log 10 for analysis. Groups were compared using two-way ANOVA followed by Bonferroni’s *post hoc* test or the Kruskal-Wallis test followed by the Mann-Whitney test. Data are presented as the mean ± standard deviation or median and minimum and maximum values. ^a^ *P* < 0.05 vs. SHAM; ^b^ *P* < 0.05 vs. SHAM OB; ^c^ *P* < 0.05 vs. UNX, ^d^ *P* < 0.05 vs. UNX OB.

FF treatment decreased TG levels in renal tissue (Figure 3a), but not cholesterol levels (Figure 3b). After 20 weeks, the SHAM OB and UNX OB groups exhibited greater TG deposition and adipocytokine levels in kidney tissue (Table 2). Meanwhile, adiponectin levels were reduced in both groups, whereas leptin levels were increased. Conversely, adiponectin levels did not change in the SHAM OB FF and UNX OB FF groups, but leptin levels were reduced (Table 2).

### Inflammatory Profile After FF Treatment

The inflammatory response triggered by excess lipids in the kidney was associated with activation of IL-6, IL-1β, MCP-1, and IFN-γ in both the SHAM OB and UNX OB groups (Table 2). In contrast, FF treatment attenuated the levels of inflammatory mediators associated with obesity or lipotoxicity, indicated by reduced expression of these cytokines in the UNX OB FF and SHAM OB FF groups (Table 2). Tissue expression of FGF-21, stimulated by PPAR-α activation, was abundant in the SHAM OB, UNX, and UNX OB groups but was reduced in groups treated with FF (Table 2).

### PAI-1 Expression and Renal Fibrosis After FF Treatment

Sirius red staining of kidney tissues viewed under polarized light revealed that renal fibrosis was slightly increased in the SHAM OB and UNX OB groups, whereas no evidence of fibrosis was observed in the FF-treated and untreated SHAM and UNX groups (Figure 3c, d). Meanwhile, the SHAM OB FF and UNX OB FF groups showed reduced PAI-1 expression compared to the other groups (Table 2).

## Discussion

The higher energy intake associated with the HFD increases the animals’ feeling of satiety, resulting in them ingesting smaller portions compared to animals fed normocaloric diets^1,25^. This behavior was also observed in the current study. Although all obesity parameters increased in the OB groups, FF treatment attenuated Lee index values and fat accumulation in the SHAM OB FF group, as observed in other studies^1,2,9^, but not in the UNX OB FF group.

The hypertrophy of the remnant kidney caused by UNX is mainly due to hemodynamic factors that trigger compensatory hypertrophy in the remnant nephrons^7^. In the current study, kidney weight and glomerular area did not change after FF treatment, corroborating results from a previous study that evaluated UNX and FF treatment without concomitant obesity^10^. However, the UNX OB groups (FF-treated or untreated) displayed increased creatinine clearance, suggesting that obesity-related kidney damage is especially severe when associated with renal mass reduction^7,26^.

Glomerular lesions (glomerulosclerosis) and albuminuria are observed in early stage kidney disease and are associated with metabolic disorders, such as reduced adiponectin levels and lipolysis and increased leptin levels and lipogenesis^27^. Moreover, renal structural and functional changes have been observed in obese kidney donors^27^. Consistent with these findings, the UNX OB group presented 10-fold greater mesangial expansion than the SHAM OB group^7,14,15^. FF treatment protected the UNX OB FF group from renal damage and reduced proteinuria and mesangial expansion, which is consistent with the results reported for obesity models without renal mass reduction^2,6,9,28^. To our knowledge, this finding has not been previously reported.

Consistent with other studies, our results support that the renal protective effect of FF is associated with decreased lipid accumulation and inflammation in the kidney^2,6^. Increased lipolysis in renal tissue is mediated by activation of PPAR-α and genes involved in lipid metabolism. Thus, FF may be used to prevent steatosis and renal lipotoxicity. However, as observed in cardiac tissues, the protective action of FF may not only depend on decreased systemic lipid levels^29^. Saturated fat intake leads to increased albuminuria and levels of inflammatory markers that cause damage in obesity models^15,25^. Indeed, the UNX OB group displayed lipid accumulation and increased expression of inflammatory cytokines associated with functional and structural kidney disorders^30^. Furthermore, obesity associated with UNX is known to upregulate MCP-1 expression and increase the number of macrophage-mediated tubulointerstitial lesions^15,16^. However, FF treatment reduced the expression of inflammatory markers MCP-1, IL-6, IL-1β, and IFN-γ in the UNX OB group, which was consistent with results reported for obese animals treated with FF^2^.

FGF-21 expression can be locally induced by lipid accumulation in renal tissue, even without correlation with plasma TG levels^5^. In the current study, the SHAM OB, UNX, and UNX OB groups exhibited abundant FGF-21 expression in renal tissue, which was significantly decreased after FF treatment. Although this finding seems paradoxical, FGF-21 tissue levels can also be affected by inflammation, and FF treatment to reduce tissue inflammation has been shown to reduce FGF-21 expression^13^. Because FF is a PPAR-α agonist and the main activator of FGF-21 in the liver, FF treatment increases FGF-21 expression in the liver and, consequently, serum levels because the liver is the main source of systemic FGF-21. However, it remains unknown whether FF exerts the same effect in renal tissue^31^. Thus, an increased abundance of circulating FGF-21 could explain its reduced expression in tissues where PPAR-α is not the main FGF-21 inducer.

PAI-1 expression is associated with oxidative stress and inflammation and is used as a prefibrotic marker^21^. Previous studies have reported that PAI-1 expression increased in obese animals, but decreased after FF treatment^2,22^. In the current study, the OB groups presented areas of renal fibrosis, which were greater in the remnant kidney of the UNX OB group, but decreased after FF treatment, as reported in the obesity model without renal mass reduction^22^. In previous obesity models, FF treatment reduced levels of oxidative stress, inflammation, and fibrosis markers^5,13^. Thus, our findings are consistent with previous reports, supporting that FF can protect the remnant kidney against obesity-induced lesions.

Although the obesity animal model used in the present study was similar to that described in other studies, only mild dyslipidemia was triggered^15,28^. This may have been due to different susceptibilities among mice populations or lower capacity of the fat source to induce metabolic changes in the study mice^1^. The measurement of circulating FGF-21 levels may support this hypothesis, but markers of lipid metabolism, such as SREBP and PPAR-α, would better characterize lipolytic activity and lipogenesis and should be investigated in future studies.

To the best of our knowledge, this is the first study to investigate the effects of FF on kidney damage caused by renal mass reduction and lipotoxicity due to obesity. The damaging effects of the HFD on the kidney were increased in UNX mice, as evidenced by higher proteinuria, mesangial expansion, renal lipid accumulation, and degree of renal fibrosis. PPAR-α activation by FF limited obesity-induced kidney inflammation and fibrosis, suggesting that FF may provide a therapeutic strategy against kidney damage caused by obesity in kidney donors.

## Supplementary Material

The following online material is available for this article:

Table S1 – High Fat Diet composition.

Table S2 – Primers and probes used for quantitative real-time PCR.

Figure S1 – Evolution of the animal’s body mass.
