# Supplementary material for: Fenofibrate attenuates renal lipotoxicity in uninephrectomized mice with high-fat diet-induced obesity
Source: J Bras Nefrol. 2024 Sep 9;46(4):e20230148. doi: 10.1590/2175-8239-JBN-2023-0148en (PMC11539900; doi:10.1590/2175-8239-JBN-2023-0148en)
Supplement: Supplementary file 1 [file 2175-8239-jbn-46-4-e20230148-s1.pdf]

Braz. J. Nephrol. (J. Bras. Nefrol.)
https://doi.org/10.1590/2175-8239-JBN-2023-0148en

Supplementary Material to “Fenofibrate attenuates renal lipotoxicity in uninephrectomized mice with high-fat diet-induced obesity”

Table S1 - High Fat Diet composition.

**Formula - High fat diet**	**g%**	**Kcal%**
Protein	*28.1*	*20*
Carbohydrate	*27.2*	*19*
Fat	*37.3*	*60*
**Total Kcal/g**	**5.625**	
	**g**	**Kcal**
Soy protein	*277*	*1,109.1*
L cistina	*3.9*	*15.6*
Corn starch	*46*	*180.7*
Dextrinized starch	*140*	*550.1*
Sucrose	*86*	*337.9*
Soybean oil	*40*	*361.9*
Pork fat	*333.5*	*3,017.6*
Mineral Mix	*12.9*	*0*
Calcium phosphate	*16.8*	*0*
Calcium carbonate	*7.1*	*0*
Potassium citrate	*21.3*	*0*
Vitamin Mix AIN93	*12.9*	*51.9*
Choline Bitartrate	*2.6*	*0*
BHT	*0.05*	*0*
	**1,000.0**	**5624.8**
