# Supplementary material for: Fenofibrate attenuates renal lipotoxicity in uninephrectomized mice with high-fat diet-induced obesity
Source: J Bras Nefrol. 2024 Sep 9;46(4):e20230148. doi: 10.1590/2175-8239-JBN-2023-0148en (PMC11539900; doi:10.1590/2175-8239-JBN-2023-0148en)
Supplement: Supplementary file 2 [file 2175-8239-jbn-46-4-e20230148-s3.pdf]

Braz. J. Nephrol. (J. Bras. Nefrol.)
https://doi.org/10.1590/2175-8239-JBN-2023-0148en

Supplementary Material to "Fenofibrate attenuates renal lipotoxicity in uninephrectomized mice with high-fat diet-induced obesity"

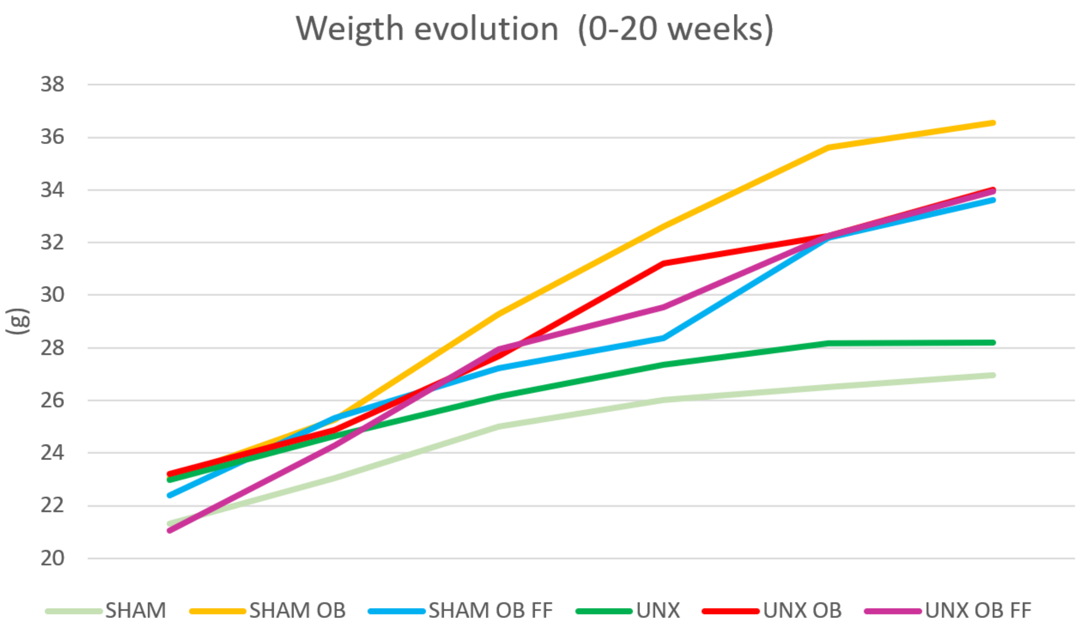

Figure S1 - Evolution of the animal's body mass.
