# Supplementary material for: Fenofibrate attenuates renal lipotoxicity in uninephrectomized mice with high-fat diet-induced obesity
Source: J Bras Nefrol. 2024 Sep 9;46(4):e20230148. doi: 10.1590/2175-8239-JBN-2023-0148en (PMC11539900; doi:10.1590/2175-8239-JBN-2023-0148en)
Supplement: Supplementary file 3 [file 2175-8239-jbn-46-4-e20230148-s2.pdf]

Braz. J. Nephrol. (J. Bras. Nefrol.)
https://doi.org/10.1590/2175-8239-JBN-2023-0148en

Supplementary Material to "Fenofibrate attenuates renal lipotoxicity in uninephrectomized mice with high-fat diet-induced obesity"

Table S2 - Primers and probes used for quantitative real-time PCR.

Target gene	Sense	Anti-sense
IL-1β	GGATGATGATGATAACCT	CATGGAGAATATCACTTGTTGG
IL-6	TCTCTGCAAGAGACTTCCATCC	AGACAGGTCTGTTGGGAGTG
MCP-1	CAAGATGATCCCAATGAGTAG	TTGGTGACAAAAACTACAGC
IFN-γ	TGAACCCTGTCGTATGCTGGGAAT	TTAGCTTTAACTCTTGGCCCAGGCA
FGF 21	CTGCGCCTACCACTGTTCC	CTGCGCCTACCACTGTTCC

IL-1β, interleukin-1β; IL-6, interleukin-6; MCP-1, monocyte chemoattractant protein-1; IFN-γ, interferon-γ; FGF-21, fibroblast growth factor-21
