# Supplementary material for: Fenofibrate attenuates renal lipotoxicity in uninephrectomized mice with high-fat diet-induced obesity
Source: J Bras Nefrol. 2024 Sep 9;46(4):e20230148. doi: 10.1590/2175-8239-JBN-2023-0148en (PMC11539900; doi:10.1590/2175-8239-JBN-2023-0148en)
Supplement: Translation

# Translation: Fenofibrato atenua a lipotoxicidade renal em camundongos uninefrectomizados com obesidade induzida por dieta rica em gordura

**DOI:** 10.1590/2175-8239-JBN-2023-0148pt

**Authors:** Barbara Bruna Abreu Castro, Petrus Ferreira Reno, Bianca Fatima Pereira, Kaique Arriel, Fabiana Bastos Bonato, Fernando Antonio Basile Colugnati, Marcos Antonio Cenedeze, Niels Olsen Saraiva-Camara, Helady Sanders-Pinheiro

**Affiliations:** 1 Universidade Federal de Juiz de Fora, Centro de Biologia da Reprodução, Núcleo de Experimentação Animal, Laboratório de Nefrologia Experimental, Juiz de Fora, MG, Brasil.; 2 Universidade Federal de Juiz de Fora, Divisão de Nefrologia, Núcleo Interdisciplinar de Estudos e Pesquisas em Nefrologia, Juiz de Fora, MG, Brasil.; 3 Universidade Federal de São Paulo, Divisão de Nefrologia, Laboratório de Imunologia Clínica e Experimental, São Paulo, SP, Brasil.; 4 Universidade de São Paulo, Instituto de Ciências Biomédicas, Departamento de Imunologia, Laboratório de Imunologia de Transplantes, São Paulo, SP, Brasil.

**Keywords:** Transtornos do Metabolismo dos Lipídeos, Obesidade, Nefrectomia, PPAR alfa, Fatores de Crescimento de Fibroblastos

## Abstract

**Introdução:**

O objetivo deste estudo foi investigar o papel do fenofibrato, um agonista do receptor ativado por proliferadores de peroxissoma-α, na lesão renal induzida por obesidade (lipotoxicidade) em camundongos submetidos à uninefrectomia.

**Métodos:**

Camundongos C57BL/6 foram submetidos a uninefrectomia e cirurgias simuladas (sham) e alimentados com dietas normocalóricas ou rica em gordura. Após 10 semanas, os camundongos obesos receberam fenofibrato a 0,02% por 10 semanas. Avaliamos função e morfologia renal, bem como níveis de mediadores inflamatórios e fibróticos e marcadores do metabolismo lipídico.

**Resultados:**

Camundongos alimentados com dieta rica em gordura desenvolveram obesidade e hiperlipidemia características, com subsequente acúmulo de lipídios e danos renais, incluindo expansão mesangial, fibrose intersticial, inflamação e proteinúria. Essas alterações foram maiores em camundongos obesos uninefrectomizados do que em camundongos obesos sham. O tratamento com fenofibrato preveniu hiperlipidemia e lesões glomerulares, reduziu o acúmulo de lipídios, melhorou a disfunção renal e atenuou a inflamação e fibrose renal. Além disso, o tratamento com fenofibrato reduziu a expressão no tecido renal do inibidor do ativador do plasminogênio-1, da proteína quimiotática de monócitos-1 e a expressão local do fator de crescimento de fibroblastos-21.

**Conclusão:**

A ativação do receptor ativado por proliferadores de peroxissoma-α pelo fenofibrato, com subsequente lipólise, atenuou lesões glomerulares e tubulointersticiais induzidas por lipotoxicidade renal, protegendo, assim, os rins de camundongos uninefrectomizados contra lesões induzidas por obesidade. Os achados do estudo sugerem uma via na ação farmacológica do fenofibrato, fornecendo insights sobre os mecanismos envolvidos no dano renal causado pela obesidade em doadores renais.

## Introdução

A obesidade e a síndrome metabólica, que são fatores de risco independentes para a doença renal crônica (DRC), estão associadas à hiperlipidemia, alterações nos níveis de adipocitocinas e aumento do estresse oxidativo, inflamação, apoptose e fibrose do tecido renal^1,2^. O recrutamento de células inflamatórias no rim leva à produção de espécies reativas de oxigênio e altera a hemodinâmica renal, induzindo a expressão do inibidor do ativador do plasminogênio-1 (PAI-1)^3^. Além disso, a lipotoxicidade renal causada pelo excesso de triglicerídeos (TG) ou ácidos graxos ativa a produção de citocinas inflamatórias e a expressão da proteína quimiotática de monócitos 1 (MCP-1) no tecido renal^1,4,5^.

A lipotoxicidade renal no rim remanescente de doadores renais obesos pode acelerar o desenvolvimento e a progressão da DRC, embora os mecanismos não estejam claros^6^. Em camundongos, a uninefrectomia (UNX) seguida de uma dieta rica em gordura (DRG) causa expansão mesangial, glomeruloesclerose e fibrose intersticial no rim remanescente^7^. A obesidade subsequente devido à DRG leva a alterações mais graves e aumenta a expressão de genes associados ao metabolismo lipídico e ao transporte de lipídios transmembrana^7^.

As terapias destinadas a diminuir os níveis séricos e teciduais de TG, a produção de EROs e a inflamação foram investigadas em modelos animais de obesidade^8^. O fenofibrato (FF), um agonista do receptor ativado por proliferadores de peroxissoma (PPAR)-α, protege contra danos renais induzidos por DRG em modelos animais^1,9^. Além disso, o FF melhorou as vias anti-inflamatórias renais dependentes de interleucina (IL)-6 em um modelo animal de UNX, embora os animais não estivessem com sobrepeso^10^. O PPAR-α regula os estoques intracelulares de lipídios^1,6,11^ e a expressão do fator de crescimento de fibroblastos-21 (FGF-21) no fígado, podendo também ser expresso em tecidos adiposos, coração e rins^12^. Um estudo que examinou os efeitos do FF em um modelo de obesidade revelou que o FGF-21 é um mediador na via de proteção contra danos renais^13^. A ativação farmacológica do PPAR-α no rim pode exercer efeitos terapêuticos por meio da lipólise, atenuando assim os efeitos deletérios da lipotoxicidade^2,11^.

O objetivo deste estudo foi determinar o papel do FF na lipotoxicidade renal associada à redução da massa renal usando um modelo de camundongo com UNX e obesidade induzida por DRG para mimetizar doadores renais que se tornam obesos.

## Métodos

### Protocolo Experimental

Camundongos C57BL/6 machos com oito semanas de idade, pesando em média 24 ± 1,6 g, foram submetidos a procedimentos SHAM ou UNX (Figura 1). No grupo SHAM, o rim esquerdo foi desencapsulado sem ser removido^14^; no grupo UNX, o rim esquerdo foi removido por meio de uma incisão abdominal^15^. A obesidade foi induzida pela administração de uma dieta rica em gordura com ingestão energética de 5.625 kcal/kg (Pragsoluções SA, Jaú, Brasil). A composição calórica da DRG foi: 28,1% de proteína, 37,3% de gordura e 27,2% de carboidratos (60% de energia proveniente da gordura total)^7^ (Table S1). A composição calórica da dieta normocalórica foi: 22% de proteína, 5% de gordura e 57% de carboidratos; energia bruta de 3.860 kcal/kg (Nuvilab, Curitiba, Brasil). O FF (Sigma-Aldrich, St. Louis, EUA) foi adicionado à dieta (0,02%, 20 mg/kg/d) das semanas 10 a 20^2,6,9^.

**Figura 1:** Desenho experimental. Abreviações – SHAM: cirurgia simulada; OB: obeso; FF: fenofibrato; UNX: uninefrectomia; DRG: dieta rica em gordura.

Às 10 e 20 semanas, os camundongos foram anestesiados por injeção intraperitoneal de xilazina (10 mg/kg) e cetamina (90 mg/kg) (Sigma-Aldrich). Amostras de sangue foram coletadas por punção cardíaca e, em seguida, os camundongos foram eutanasiados por ruptura do diafragma sendo o rim direito removido para análise.

Todos os procedimentos envolvendo animais foram realizados de acordo com os padrões éticos estabelecidos pela Comissão de Ética no Uso de Animais da Universidade Federal de Juiz de Fora (número de aprovação IRB 046/2018).

### Ingestão Dietética e Alterações na Obesidade

A ingestão de ração e o peso total dos animais foram medidos (g) a cada 4 semanas (Figure S1) e calculou-se a ingestão média diária. Como medida das alterações induzidas pela DRG, avaliamos o peso total, o ganho de peso, a gordura total e o índice de Lee às 10 e 20 semanas. Consideramos gordura total como sendo os tecidos adiposos retroperitoneal e epididimal removidos e pesados durante a eutanásia^7,14,16^. O comprimento nasoanal foi medido (cm) e utilizado para calcular o índice de Lee^17^.

### Análise de Sangue e Urina

Os camundongos foram mantidos em gaiolas metabólicas por 24 horas para coleta de urina, e a proteinúria de 24 horas foi determinada (mg/24 h) com o uso de um kit Sensiprot Liquiform (Labtest, Lagoa Santa, Brasil). Os níveis séricos de creatinina foram medidos usando o ensaio Creatinina K Liquiform (Labtest) para calcular o *clearance* de creatinina. O *clearance* de creatinina foi calculado como a excreção urinária de creatinina de 24 horas (mg) dividida pela creatinina sérica (mg/dL), multiplicada por 1440 (U×V/P×1440) e expressa em mL/min^7,14^. Os níveis séricos de TG e colesterol total foram medidos usando um analisador Cobas (Roche Diagnostics, Basel, Suíça) na semana 20.

### Extração de Lipídios e Conteúdo Lipídico Renal

Os lipídios foram extraídos por meio da homogeneização de 35 mg de tecido renal com 500 μL de uma solução de clorofórmio/metanol e água, seguida de centrifugação a 3.000 rpm por 5 minutos. A camada inferior foi transferida para um novo tubo. Após a evaporação do líquido, 100 μL de álcool isopropílico foram adicionados à amostra para solubilização dos lipídios^1^. Os níveis de TG e colesterol foram determinados usando um teste enzimático colorimétrico *Liquiform Cholesterol and Triglycerides (Labtest)* nas semanas 10 e 20.

### Análise Histológica

Metade do rim direito foi fixada em formalina a 10% para análise histológica^7,14,18–20^. As amostras de tecido renal foram obtidas de cinco animais por grupo de tratamento. As seções (5 μm) foram coradas com hematoxilina-eosina (H&E) e sirius red (Sigma-Aldrich). As lâminas foram examinadas e fotografadas utilizando um microscópio Axio Scope A1 (Carl Zeiss, Göttingen, Alemanha) acoplado a uma câmera digital para microscópio, com o software do sistema AmScope MU1000 (AmScope, Irvine, TX, EUA).

Foram realizadas análises semiquantitativas e quantitativas da expansão mesangial. Para avaliar a expansão mesangial de forma semiquantitativa, foram analisadas as lâminas de 5 animais de cada grupo. Dez glomérulos com polos vasculares por lâmina foram fotografados com ampliação de 400×. Cada glomérulo foi analisado qualitativamente quanto à expansão mesangial, definida como o espaço mesangial excedendo a largura de duas células mesangiais por pelo menos dois lobos glomerulares, sendo classificado como “Ausência” ou “Presença” de expansão mesangial^18,19^. Os resultados foram expressos como a porcentagem de glomérulos com expansão mesangial por lâmina.

A análise quantitativa foi realizada com o software ImageJ 1.52n (*National Institutes of Health*, Bethesda, MD, EUA). Foram analisadas as lâminas de 5 animais de cada grupo. Dez glomérulos com polos vasculares por lâmina foram fotografados com ampliação de 400×, o que corresponde a uma área de 0,045 mm^2^. Em cada fotografia, toda a área ocupada pelo tufo glomerular foi marcada. A medição da área ocupada na imagem forneceu valores numéricos expressos em pixels. Essa variável foi denominada área glomerular^21^.

A fibrose renal foi quantificada via coloração de sirius red sob luz polarizada^20^. Foram tiradas dez fotomicrografias por lâmina com ampliação de 200×. As áreas em vermelho brilhante foram quantificadas com o software ImageJ 1.52n. Os resultados foram expressos como a porcentagem da área intersticial de fibrose por córtex/área cortical total. Em seguida, calculou-se a média da área de coloração positiva para cada lâmina^21^.

### Adipocitocinas e Fator pré-Fibrótico PAI-1

Aproximadamente 35 mg de tecido foram macerados em 1 mL de tampão de lise RIPA a 4°C, e a concentração de proteína foi medida usando o kit de ensaio de proteínas Micro BCA (*Thermo Fisher Scientific*, Waltham, MA, EUA). As concentrações de adiponectina, leptina e fator pré-fibrótico PAI-1 presentes na amostra foram analisadas utilizando o ensaio *MILLIPLEX MAP Mouse Adipocyte Magnetic Panel - Endocrine Multiplex Assay* (Merck, Barueri, Brasil). O teste foi realizado usando o sistema Bio-Plex 200 com o software Bio-Plex Manager versão 5.0 (Bio-Rad, Hercules, CA, EUA)^22^.

### Mediadores Inflamatórios e Fibróticos Avaliados por PCR Quantitativa em Tempo Real

O sistema de amplificação TaqMan (*Applied Biosystems,* Branchburg, NJ, EUA) foi utilizado para realizar a PCR quantitativa em tempo real (qRT-PCR) com o termociclador 7500 *Real Time PCR System* (*Applied Biosystems*, Cingapura). Todas as amostras foram analisadas em triplicata. Foi usada uma relação comparativa entre os ciclos de reação (CT) para determinar a expressão do gene alvo em relação ao gene controle hipoxantina-guanina fosforibosiltransferase^23^. Primers e sondas sintetizados para IL-6, IL-1β, MCP-1, IFN-γ e FGF-21 foram utilizados para avaliar o efeito do FF no perfil inflamatório (produtos de expressão gênica *Assays-On-Demand*; *Applied Biosystems*, Foster City, CA, EUA) (Table S2). Os valores de CT dos genes alvo foram normalizados para seus respectivos genes controle para cada amostra, e o valor resultante foi utilizado para demonstrar a expressão relativa dos genes alvo por meio do método 2-ΔΔCT, conforme descrito anteriormente^24^.

### Análise Estatística

Os testes de Kolmogorov-Smirnov e Shapiro-Wilk foram utilizados para avaliar a distribuição das variáveis. A homogeneidade de variância foi verificada por meio do teste de Levene. A reprodutibilidade das lesões nos modelos experimentais (obesidade e UNX) foi avaliada na semana 10 utilizando a análise de variância unidirecional (ANOVA), seguida pelo teste *post-hoc* de Bonferroni. Os efeitos da UNX, da dieta e do tratamento com FF sobre os parâmetros avaliados foram verificados na semana 20 com um modelo linear geral para comparar grupos usando ANOVA de duas vias, seguido pelo teste *post-hoc* de Bonferroni. Algumas variáveis foram transformadas em valores de log 10 para obter distribuição normal e homogeneidade de variância. O teste não paramétrico de Kruskal-Wallis, seguido pelo teste de Mann-Whitney, foi utilizado para variáveis que não atenderam aos critérios de normalidade e homogeneidade para comparação entre grupos. A significância estatística foi definida como P < 0,05. A análise estatística foi realizada com o software SPSS versão 15.0 (SPSS Inc., Chicago, IL, EUA). Os dados utilizados na análise estão disponíveis mediante solicitação no *Open Science Framework*, https://osf.io/s3j9a.

## Resultados

### Avaliação de Camundongos do Modelo UNX e de Obesidade na Semana 10

O modelo de obesidade com disfunção renal foi induzido com sucesso pela DRG após 10 semanas (Tabela 1). O grupo UNX apresentou pesos renais mais elevados do que o grupo SHAM, sem perda da função renal (Tabela 1). O grupo UNX OB apresentou menor ingestão dietética, maior acúmulo de gordura, valores mais altos do índice de Lee e maior proteinúria e hiperfiltração do que o grupo UNX (Tabela 1).

**Tabela 1:** Parâmetros metabólicos e de função renal de camundongos submetidos a cirurgias SHAM ou UNX e alimentados com dietas normocalórica ou rica em gordura por 10 semanas. os dados são apresentados como média ± desvio padrão

Parâmetros	10 semanas			
Grupo (N)	SHAM (N = 9)	SHAM OB (N = 10)	UNX (N = 8)	UNX OB (N = 8)
Ingestão (g/d)	3,6 ± 0,7	2,2 ± 0,3^a^	4,2 ± 0,7^b^	2,6 ± 0,3^a c^
Peso total (g)	27,2 ± 1,1	29,8 ± 3,3	25,2 ± 0,9^b^	27,7 ± 2,2
Ganho de peso (g)	3,0 ± 2,0	6,5 ± 1,9^a^	3,4 ± 1,8^b^	4,4 ± 2,1
Gordura total (g)	0,41 ± 0,17	1,46 ± 0,66^a^	0,19 ± 0,08^b^	1,03 ± 0,38^a c^
Peso renal (g)	0,17 ± 0,01	0,15 ± 0,01	0,20 ± 0,03^a b^	0,20 ± 0,01^b^
Índice de Lee (g/cm^3^)	310 ± 6	318 ± 6^a^	306 ± 5^b^	308 ± 6^b^
**Grupo (N)**	**SHAM (N = 3)**	**SHAM OB (N = 3)**	**UNX (N = 3)**	**UNX OB (N = 3)**
Proteinúria (mg/24 h)	2,1 ± 1,2	9,0 ± 2,6^a^	1,0 ± 1,1^b^	10,9 ± 1,5^a c^
Creatinina sérica (mg/dL)	1,0 ± 0,1	0,9 ± 0,1	0,9 ± 0,1	0,9 ± 0,1
Clearance de creatinina (mL/min)	0,06 ± 0,04	0,25 ± 0,09	0,04 ± 0,03^b^	0,34 ± 0,07^a c^
**Grupo (N)**	**SHAM (N = 5)**	**SHAM OB (N = 5)**	**UNX (N = 5)**	**UNX OB (N = 4)**
TG no rim (mg/g tecido)	2,91 ± 0,87	4,06 ± 0,25	3,29 ± 0,25	4,56 ± 1,40
**Grupo (N)**	**SHAM (N = 4)**	**SHAM OB (N = 4)**	**UNX (N = 4)**	**UNX OB (N = 4)**
Colesterol nos rins (mg/g tecido)^log^	3,60 ± 0,25	3,51 ± 0,24	3,83 ± 0,24	4,67 ± 1,18

Abreviações – SHAM: cirurgia simulada; OB: obeso; UNX: uninefrectomia; DRG: dieta rica em gordura; TG: triglicerídeos.

Notas – ^log^Variáveis foram transformadas em log de base 10 para análise. Os grupos foram comparados usando ANOVA seguido do teste *post-hoc* de Bonferroni.

^a^ *P* < 0,05 vs. SHAM,

^b^ *P* < 0,05 vs. SHAM OB,

^c^ *P* < 0,05 vs. UNX.

### Avaliação do Modelo de Obesidade Após o Tratamento com FF

Ao final do experimento, os grupos SHAM OB e UNX OB (grupos alimentados com DRG) apresentaram menor ingestão dietética e maiores alterações associadas à obesidade do que os grupos SHAM e UNX (Tabela 2).

**Tabela 2:** Parâmetros metabólicos, perfis lipídicos, níveis de adipocitocinas e marcadores inflamatórios e expressão de PAI-1 em camundongos submetidos a cirurgias SHAM e UNX, alimentados com dietas normocalórica ou rica em gordura e tratados com FF por 10 semanas. os dados são apresentados como média ± desvio padrão ou mediana e valores mínimo e máximo.

Parâmetros	20 semanas					
Grupo (N)	SHAM (N = 18)	SHAM OB (N = 19)	SHAM OB FF (N = 17)	UNX (N = 19)	UNX OB (N = 18)	UNX OB FF (N = 17)
Ingestão (g/d)	3,48 ± 0,45	2,51 ± 0,24^a^	2,68 ± 0,34^a^	3,47 ± 0,42	2,51 ± 0,30^c^	2,69 ± 0,55^c^
Peso total (g)	27,2 (24,5 – 30,0)	35,5 (29,5 – 46,5)^a^	33,5 (26,0 – 44,0)^a^	28,0 (25,0 – 31,5)	33,2 (25,0 – 46,0)^c^	32,0 (26,5 – 47,5)^c^
Ganho de peso (g)log	5,67 ± 1,12	13,53 ± 4,21^a^	9,55 ± 5,35^a^	5,24 ± 2,06	10,81 ± 5,65^c^	10,95 ± 6,22^c^
Gordura total (g)	0,35 (0,17 – 0,51)	2,49 (0,76 – 3,30)^a^	1,56 (0,47 – 2,56)^a b^	0,34 (0,20 – 0,71)	1,62 (0,35 – 3,38)^c^	2,00 (0,70 – 3,24)^c^
Peso renal (g)log	0,17 ± 0,02^c d^	0,16 ± 0,02^c d^	0,18 ± 0,01^c d^	0,23 ± 0,02^a b^	0,22 ± 0,02^a b^	0,22 ± 0,02^a b^
Índice de Lee (g/cm3)	305 (296 – 321)	319 (305 – 342)^a^	313 (299 – 328)^a b^	302 (292 – 312)	315 (292 – 332)^c^	318 (305 – 344)^c^
**Grupo (N)**	**SHAM** **(N = 9)**	**SHAM OB** **(N = 10)**	**SHAM OB FF** **(N = 10)**	**UNX** **(N = 10)**	**UNX OB** **(N = 10)**	**UNX OB FF** **(N = 9)**
TG sérico (mg/dL)log	89,9 ± 25,4	102,7 ± 37,2	58,0 ± 17,1^a b^	79,5 ± 14,6	113,9 ± 51,4	79,4 ± 21,2^c d^
**Grupo (N)**	**SHAM** **(N = 9)**	**SHAM OB** **(N = 10)**	**SHAM OB FF** **(N = 10)**	**UNX** **(N = 9)**	**UNX OB** **(N = 10)**	**UNX OB FF** **(N = 9)**
Colesterol sérico (mg/dL)log	83,4 ± 11,2	185,0 ± 15,0^a^	213,9 ± 14,5^a b^	95,2 ± 8,5^a^	194,5 ± 35,1^c^	229,7 ± 17,1^c d^
**Grupo (N)**	**SHAM** **(N = 11)**	**SHAM OB** **(N = 11)**	**SHAM OB FF** **(N = 11)**	**UNX** **(N = 11)**	**UNX OB** **(N = 12)**	**UNX OB FF** **(N = 11)**
Adiponectina (pg/mg proteína)	7,12 ± 1,51	4,93 ± 1,71^a^	6,26 ± 1,02^a^	7,08 ± 1,54	5,45 ± 1,22^c^	5,75 ± 1,06^c^
Leptina (pg/mg proteína)log	0,08 ± 0,02	0,17 ± 0,05^a^	0,14 ± 0,06^a b^	0,09 ± 0,03	0,30 ± 0,15^c^	0,20 ± 0,07^c d^
PAI-1 (pg/mg proteína)	0,011 ± 0,002	0,015 ± 0,005	0,012 ± 0,002^b^	0,015 ± 0,005	0,017 ± 0,006	0,011 ± 0,003^d^
**Grupo (N)**	**SHAM** **(N = 10)**	**SHAM OB** **(N = 10)**	**SHAM OB FF** **(N = 10)**	**UNX** **(N = 10)**	**UNX OB** **(N = 10)**	**UNX OB FF** **(N = 10)**
IL1-B (2^△△CT^)	0,71 (0,46 – 1,29)	3,49 (2,44 – 4,42)^a^	0,74 (0,49 – 1,68)^b^	3,48 (2,53 – 7,08)	3,78 (2,01 – 10,90)	0,64 (0,37 – 1,00)^c d^
MCP-1 (2^△△CT^)^log^	1,25 ± 0,58	2,48 ± 0,96^a^	1,10 ± 0,46^a b^	2,71 ± 0,61	3,41 ± 1,36^c^	0,99 ± 0,23^c d^
IFN-g (2^△△CT^)	0,84 ± 0,19	2,21 ± 0,39^a^	1,34 ± 0,21^b^	1,96 ± 0,35	2,99 ± 0,53^c^	1,77 ± 0,37^d^
**Grupo (N)**	**SHAM** **(N = 8)**	**SHAM OB** **(N = 6)**	**SHAM OB FF** **(N = 9)**	**UNX** **(N = 3)**	**UNX OB** **(N = 7)**	**UNX OB FF** **(N = 10)**
IL -6 (2^△△CT^)^log^	0,54 ± 0,34	1,66 ± 0,83^a^	1,15 ± 0,93^b^	0,83 ± 0,17	2,62 ± 1,88^c^	0,51 ± 0,34^d^
**Grupo (N)**	**SHAM** **(N = 9)**	**SHAM OB** **(N = 10)**	**SHAM OB FF** **(N = 9)**	**UNX** **(N = 10)**	**UNX OB** **(N = 8)**	**UNX OB FF** **(N = 8)**
FGF-21 (2^△△CT^)^log^	0,71 ± 0,50	5,37 ± 3,60^a^	0,69 ± 0,51^a b^	7,28 ± 3,93	10,67 ± 10,44^c^	0,40 ± 0,38^c d^

Abreviações – SHAM: cirurgia simulada; OB: obeso; FF: fenofibrato; UNX: uninefrectomia; TG: triglicerideos; IL -1β: interleucina-1β; IL -6: interleucina-6; MCP-1: proteína quimiotática de monócitos-1; IFN-γ: interferon-γ; FGF -21: fator de crescimento de fibroblastos-21; PAI-1: inibidor do ativador do plasminogênio-1.

Notas – ^log^Variáveis transformadas em log de base 10 para análise. Os grupos foram comparados utilizando ANOVA de duas vias seguido do teste *post hoc* de Bonferroni ou o teste de Kruskal-Wallis, ao qual se seguiu o teste de Mann-Whitney.

^a^ *P* < 0,05 vs. SHAM;

^b^ *P* <0,05 vs. SHAM OB;

^c^ *P* < 0,05 vs. UNX,

^d^ *P* < 0,05 vs. UNX OB.

O grupo SHAM OB FF apresentou menor acúmulo de gordura e valores do índice de Lee em comparação com o grupo SHAM OB (Tabela 2). Em contraste, o grupo UNX OB FF não apresentou diferença nos parâmetros de obesidade em comparação com o grupo UNX OB (Tabela 2). Além disso, os grupos UNX exibiram pesos renais maiores do que os grupos SHAM (Tabela 2). O tratamento com FF não teve efeito sobre a ingestão dietética nos grupos SHAM OB FF ou UNX OB FF, embora tenha evitado o acúmulo de gordura no grupo SHAM OB FF, indicado por um valor de índice de Lee menor do que no grupo SHAM (Tabela 2).

### Perfil Lipídico Após o Tratamento com FF

Foi demonstrado que o FF reduz os níveis séricos de TG; portanto, avaliamos o perfil lipídico do modelo UNX OB. Embora os níveis de TG não tenham sido significativamente mais elevados nos grupos SHAM OB e UNX OB, o tratamento com FF reduziu os níveis de TG nos grupos SHAM OB FF e UNX OB FF. Os níveis de colesterol não se alteraram significativamente após o tratamento com FF (Tabela 2).

### Função Renal e Glomerulopatia Após Tratamento com FF

A indução da obesidade nos grupos SHAM OB e UNX OB foi associada ao aumento da proteinúria (Figura 2a). No entanto, o tratamento com FF atenuou a progressão das lesões induzidas por obesidade ou lipotoxicidade, conforme indicado pela redução da proteinúria no grupo UNX OB FF (Figura 2a). A obesidade não aumentou os níveis séricos de creatinina (Figura 2b). O clearance de creatinina foi maior nos grupos UNX, UNX OB e UNX OB FF do que nos grupos SHAM, SHAM OB e SHAM OB FF. A obesidade causou hiperfiltração, a qual não melhorou com o tratamento com FF (Figura 2c).

**Figura 2:** Avaliação da função renal (a, b, c) e morfologia glomerular (d, e, f: H&E, 400×) após indução de obesidade e tratamento com FF (10 semanas). Abreviações – SHAM: cirurgia simulada; OB: obeso; FF: fenofibrato; UNX: uninefrectomia; HE: hematoxilina-eosina. Notas – ^log^Variáveis transformadas em log na base 10 para análise. Os grupos foram comparados utilizando ANOVA de duas vias seguido do teste *post hoc* de Bonferroni ou o teste de Kruskal-Wallis, ao qual se seguiu o teste de Mann-Whitney. Os dados são apresentados como média ± desvio padrão ou mediana e valores mínimo e máximo. ^a^ *P* < 0,05 vs. SHAM; ^b^ *P* < 0,05 vs. SHAM OB; ^c^ *P* < 0,05 vs. UNX, ^d^ *P* < 0,05 vs. UNX OB.

A coloração H&E dos tecidos renais revelou que a área glomerular aumentou nos grupos UNX, independentemente da obesidade, que não sofreu alteração pelo tratamento com FF (Figura 2d). A porcentagem de glomérulos com expansão mesangial nos grupos SHAM OB e UNX OB foi maior do que nos grupos SHAM e UNX, respectivamente (Figura 2e). Os grupos SHAM OB FF e UNX OB FF apresentaram reduções de 50% e 70% na expansão mesangial em comparação com os grupos SHAM OB e UNX OB, respectivamente (Figura 2e e f).

### Depósitos Lipídicos nos Rins e Níveis de Adipocitocina Após Tratamento com FF

O tratamento com FF diminuiu os níveis de TG no tecido renal (Figura 3a), mas não os níveis de colesterol (Figura 3b). Após 20 semanas, os grupos SHAM OB e UNX OB apresentaram maior deposição de TG e níveis de adipocitocina no tecido renal (Tabela 2). Enquanto isso, os níveis de adiponectina foram reduzidos em ambos os grupos, enquanto os níveis de leptina aumentaram. Por outro lado, os níveis de adiponectina não se alteraram nos grupos SHAM OB FF e UNX OB FF, mas os níveis de leptina foram reduzidos (Tabela 2).

**Figura 3:** Valores de deposição de lipídios no tecido renal (a, b) e fibrose intersticial renal (c: Sirius red, 200×, D) após indução de obesidade e tratamento com FF (10 semanas). Abreviações – SHAM: cirurgia simulada; OB: obeso; FF: fenofibrato; UNX: uninefrectomia; TG: triglicerídeos. Notas – ^log^Variáveis transformadas em log na base 10 para análise. Os grupos foram comparados utilizando ANOVA de duas vias seguido do teste *post hoc* de Bonferroni ou o teste de Kruskal-Wallis, ao qual se seguiu o teste de Mann-Whitney. Os dados são apresentados como média ± desvio padrão ou mediana e valores mínimo e máximo. ^a^ *P* < 0,05 vs. SHAM; ^b^ *P* < 0,05 vs. SHAM OB; ^c^ *P* < 0,05 vs. UNX, ^d^ *P* < 0,05 vs. UNX OB.

### Perfil Inflamatório Após Tratamento com FF

A resposta inflamatória desencadeada pelo excesso de lipídios no rim foi associada à ativação de IL-6, IL-1β, MCP-1 e IFN-γ tanto no grupo SHAM OB quanto no UNX OB (Tabela 2). Por outro lado, o tratamento com FF atenuou os níveis de mediadores inflamatórios associados à obesidade ou lipotoxicidade, indicado pela redução da expressão dessas citocinas nos grupos UNX OB FF e SHAM OB FF (Tabela 2). A expressão tecidual de FGF-21, estimulada pela ativação do PPAR-α, foi abundante nos grupos SHAM OB, UNX e UNX OB, mas foi reduzida nos grupos tratados com FF (Tabela 2).

### Expressão do PAI-1 e Fibrose Renal Após Tratamento com FF

A coloração com sirius red dos tecidos renais, visualizada sob luz polarizada, revelou que a fibrose renal aumentou ligeiramente nos grupos SHAM OB e UNX OB, enquanto nenhuma evidência de fibrose foi observada nos grupos SHAM e UNX tratados e não tratados com FF (Figura 3c, d). Enquanto isso, os grupos SHAM OB FF e UNX OB FF apresentaram expressão reduzida de PAI-1 em comparação aos demais grupos (Tabela 2).

## Discussão

A maior ingestão energética associada à DRG aumenta a sensação de saciedade dos animais, resultando na ingestão de porções menores em comparação com os animais alimentados com dietas normocalóricas^1,25^. Esse comportamento também foi observado no presente estudo. Embora todos os parâmetros de obesidade tenham aumentado nos grupos OB, o tratamento com FF atenuou os valores do índice de Lee e o acúmulo de gordura no grupo SHAM OB FF, conforme observado em outros estudos^1,2,9^, mas não no grupo UNX OB FF.

A hipertrofia do rim remanescente causada pela UNX se deve principalmente a fatores hemodinâmicos que desencadeiam hipertrofia compensatória nos néfrons remanescentes^7^. No presente estudo, o peso renal e a área glomerular não se alteraram após o tratamento com FF, corroborando os resultados de um estudo anterior que avaliou o tratamento com UNX e FF sem obesidade concomitante^10^. Entretanto, os grupos UNX OB (tratados ou não com FF) apresentaram aumento do *clearance* de creatinina, sugerindo que o dano renal relacionado à obesidade é especialmente grave quando associado à redução de massa renal^7,26^.

Lesões glomerulares (glomeruloesclerose) e albuminúria são observadas no estágio inicial da doença renal e estão associadas a distúrbios metabólicos, como redução dos níveis de adiponectina e lipólise e aumento dos níveis de leptina e lipogênese^27^. Além disso, foram observadas alterações estruturais e funcionais no rim de doadores renais obesos^27^. Em consonância com esses achados, o grupo UNX OB apresentou expansão mesangial 10 vezes maior do que o grupo SHAM OB^7,14,15^. O tratamento com FF protegeu o grupo UNX OB FF contra danos renais e reduziu a proteinúria e a expansão mesangial, o que é consistente com os resultados relatados para modelos de obesidade sem redução de massa renal^2,6,9,28^. Até onde sabemos, esse achado não foi relatado anteriormente.

Em consonância com outros estudos, nossos resultados sustentam que o efeito protetor renal do FF está associado à redução do acúmulo de lipídios e da inflamação nos rins^2,6^. O aumento da lipólise no tecido renal é mediado pela ativação do PPAR-α e dos genes envolvidos no metabolismo lipídico. Assim, o FF pode ser utilizado para prevenir esteatose e lipotoxicidade renal. Entretanto, conforme observado nos tecidos cardíacos, a ação protetora do FF pode não depender somente da redução dos níveis lipídicos sistêmicos^29^. A ingestão de gordura saturada leva ao aumento da albuminúria e dos níveis de marcadores inflamatórios que causam danos em modelos de obesidade^15,25^. De fato, o grupo UNX OB apresentou acúmulo lipídico e aumento da expressão de citocinas inflamatórias associadas a distúrbios renais funcionais e estruturais^30^. Além disso, sabe-se que a obesidade associada à UNX eleva a expressão de MCP-1 e aumenta o número de lesões tubulointersticiais mediadas por macrófagos^15,16^. No entanto, o tratamento com FF reduziu a expressão dos marcadores inflamatórios MCP-1, IL-6, IL-1β e IFN-γ no grupo UNX OB, o que foi consistente com os resultados relatados para animais obesos tratados com FF^2^.

A expressão do FGF-21 pode ser induzida localmente pelo acúmulo de lipídios no tecido renal, mesmo sem correlação com os níveis plasmáticos de TG^5^. No presente estudo, os grupos SHAM OB, UNX e UNX OB apresentaram expressão abundante de FGF-21 no tecido renal, que foi significativamente reduzida após o tratamento com FF. Embora esse achado pareça paradoxal, os níveis teciduais de FGF-21 também podem ser afetados pela inflamação, e o tratamento com FF para reduzir a inflamação tecidual demonstrou reduzir a expressão de FGF-21^13^. Como o FF é um agonista do PPAR-α e o principal ativador do FGF-21 no fígado, o tratamento com FF aumenta a expressão do FGF-21 no fígado e, consequentemente, os níveis séricos, uma vez que o fígado é a principal fonte de FGF-21 sistêmico. Entretanto, ainda não se sabe se o FF exerce o mesmo efeito no tecido renal^31^. Assim, uma maior abundância de FGF-21 circulante pode explicar sua expressão reduzida em tecidos nos quais o PPAR-α não é o principal indutor de FGF-21.

A expressão do PAI-1 está associada ao estresse oxidativo e à inflamação e é utilizada como um marcador pré-fibrótico^21^. Estudos anteriores relataram que a expressão do PAI-1 aumentou em animais obesos, mas diminuiu após o tratamento com FF^2,22^. No presente estudo, os grupos OB apresentaram áreas de fibrose renal, que foram maiores no rim remanescente do grupo UNX OB, mas diminuíram após o tratamento com FF, conforme relatado no modelo de obesidade sem redução da massa renal^22^. Em modelos anteriores de obesidade, o tratamento com FF reduziu os níveis de estresse oxidativo, inflamação e marcadores de fibrose^5,13^. Assim, nossos achados são consistentes com relatos anteriores, sustentando o fato de que o FF pode proteger o rim remanescente contra lesões induzidas pela obesidade.

Embora o modelo animal de obesidade utilizado no presente estudo tenha sido semelhante ao descrito em outros estudos, apenas uma dislipidemia leve foi desencadeada^15,28^. Isso pode ter ocorrido devido a diferentes suscetibilidades entre as populações de camundongos ou à menor capacidade da fonte de gordura de induzir alterações metabólicas nos camundongos do estudo^1^. A medição dos níveis circulantes de FGF-21 pode apoiar essa hipótese, mas marcadores do metabolismo lipídico, como SREBP e PPAR-α, caracterizariam melhor a atividade lipolítica e a lipogênese e devem ser investigados em estudos futuros.

De acordo com nosso conhecimento, este é o primeiro estudo a investigar os efeitos do FF nos danos renais causados pela redução da massa renal e lipotoxicidade devido à obesidade. Os efeitos prejudiciais da DRG nos rins foram aumentados em camundongos UNX, conforme evidenciado por maior proteinúria, expansão mesangial, acúmulo renal de lipídios e grau de fibrose renal. A ativação do PPAR-α pelo FF limitou a inflamação e fibrose renal induzidas pela obesidade, sugerindo que o FF pode fornecer uma estratégia terapêutica contra os danos renais causados pela obesidade em doadores renais.

## Material Suplementar

O seguinte material online está disponível para o presente artigo:

Table S1 – High Fat Diet composition.

Table S2 – Primers and probes used for quantitative real-time PCR.

Figure S1 – Evolution of the animal’s body mass.
